# Isolation, Characterization and Biological Action of Type-1 Ribosome-Inactivating Proteins from Tissues of *Salsola soda* L.

**DOI:** 10.3390/toxins14080566

**Published:** 2022-08-19

**Authors:** Nicola Landi, Sara Ragucci, Lucía Citores, Angela Clemente, Hafiza Z. F. Hussain, Rosario Iglesias, José M. Ferreras, Antimo Di Maro

**Affiliations:** 1 Department of Environmental, Biological and Pharmaceutical Sciences and Technologies (DiSTABiF), University of Campania Luigi Vanvitelli, Via Vivaldi 43, 81100 Caserta, Italy; 2 Department of Biochemistry and Molecular Biology and Physiology, Faculty of Sciences, University of Valladolid, E-47011 Valladolid, Spain

**Keywords:** antifungal activity, agretti, cytotoxicity, edible plants, protein purification, rRNA N-glycosylases

## Abstract

Ribosome-inactivating proteins (RIPs) are known as RNA N-glycosylases. They depurinate the major rRNA, damaging ribosomes and inhibiting protein synthesis. Here, new single-chain (type-1) RIPs named sodins were isolated from the seeds (five proteins), edible leaves (one protein) and roots (one protein) of *Salsola soda* L. Sodins are able to release Endo’s fragment when incubated with rabbit and yeast ribosomes and inhibit protein synthesis in cell-free systems (IC_50_ = 4.83–79.31 pM). In addition, sodin 5, the major form isolated from seeds, as well as sodin eL and sodin R, isolated from edible leaves and roots, respectively, display polynucleotide:adenosine glycosylase activity and are cytotoxic towards the Hela and COLO 320 cell lines (IC_50_ = 0.41–1200 nM), inducing apoptosis. The further characterization of sodin 5 reveals that this enzyme shows a secondary structure similar to other type-1 RIPs and a higher melting temperature (Tm = 76.03 ± 0.30 °C) and is non-glycosylated, as other sodins are. Finally, we proved that sodin 5 possesses antifungal activity against *Penicillium digitatum*.

## 1. Introduction

Ribosome-inactivating proteins (RIPs) are a group of ribotoxic enzymes which act on ribosomes in a highly specific and irreversible manner. They are N-glycosylases (EC 3.2.2.22) capable of hydrolyzing the N-glycosidic bond of a specific adenosine in the sarcin ricin loop (SRL) of major rRNA (A_4324_, rat liver 28S rRNA numbering) [1]. The consequent formation of an apurinic site prevents the interaction of prokaryotic or eukaryotic elongation factors (EF-G or EF-2, respectively) with ribosomes, blocking mRNA-tRNA translocation and thus inhibiting protein synthesis and inducing the apoptotic pathway [2]. In addition, these enzymes possess polynucleotide:adenosine glycosylase (PNAG) activity on different polynucleotide substrates (e.g., viral RNA and herring sperm DNA) [3,4,5]. On the other hand, other enzymatic activities such as DNAse [6,7], RNAse [8], chitinase [9], phosphatase, lipase [10] and superoxide dismutase properties [11,12,13] have also been attributed to RIPs, although some authors ascribe these activities to possible contamination [4,14]. RIPs are mostly found in flowering plants [15,16], few are found in fungi [17] and bacteria [18] and one is found in alga [19].

Based on the presence or absence of a quaternary structure, there are two main groups of RIPs: single-chain proteins (type-1 RIPs), with a molecular weight of ~30 kDa and basic pI, and two-chain proteins (type-2 RIPs), with a molecular weight of ~60 kDa and neutral pI. The latter consist of an enzymatic active A-chain linked through a disulphide bridge to a lectinic B-chain, which allows for the entry into the cell. For this reason, type-2 RIPs are more toxic with respect to type-1 RIPs. In addition, a third group of non-canonical single-chain RIPs (type-3 RIPs) was found only in Poaceae, including JIP60 isolated from barley [20,21] and b-32 isolated from maize [22], made of a type-1-like N-terminal domain with N-glycosylase activity, covalently linked to a C-terminal domain with an unknown function [23].

Although their physiological role is still unknown, RIPs have a broad spectrum of activities, including antiviral, antibacterial and antifungal action, as well as anticancer properties [24,25,26]. Thus, the potential applications of RIPs span many fields, from agriculture (e.g., toxicity to pests and antifungal activity) [27] to biomedicine for the construction of antibody-RIPs conjugates (i.e., immunotoxins) against target cancer cells [28]. From the foregoing, it is clear that continuing the study of RIPs distribution in higher plants (including edible species) can contribute to expanding the availability on novel potential biotechnological and pharmacological tools.

*Salsola soda* L., commonly known as barilla plant or ‘agretti’ in Italy, is an annual, edible halophytic plant that is widespread in south Europe, mostly near the coast. In the past, the plant was used for the production of an impure sodium carbonate named ‘barilla’ from the sodium chloride in the soil (e.g., to make soap and glass) [29]. The plant tissues are rich in alkaloids, saponins, coumarins and sterols, as well as flavonoids with anti-inflammatory and antidiabetic potential [30]. According to the taxonomy, *S. soda* belongs to Caryophyllales [31], a plant order known as a source of RIPs [32]. The plant is an Amaranthacea with succulent leaves, small sessile hermaphrodite flowers and indehiscent fruits. It is known that the isoforms of these ribonucleolytic enzymes can be found in different tissues of the same plant [33,34,35] or in the same plant tissue [36]. However, little information about the distribution of RIPs in plant organs and tissues is available in the literature, especially with regard to edible plants, considering that they are often consumed raw [37].

In this framework, we report the purification and characterization of seven novel RIPs named sodins: five from the seeds, one from the roots and one from the edible leaves of *S. soda*. We describe the distribution of both N-glycosylase and PNAG activity in the different tissues of *S. soda*. Moreover, considering the high expression of sodins in the seeds, we further characterized the major form (i.e., sodin 5) by evaluating some structural features, the cytotoxic effect in cancer cell lines and the antifungal activity against *Penicillium digitatum*.

## 2. Results and Discussion

### 2.1. Purification of Type-1 RIPs from Salsola soda Seeds

**Figure 1:**
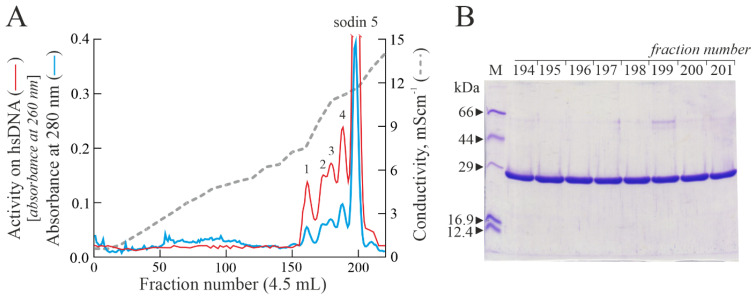
(**A**) Elution profile after cation exchange chromatography on the CM-Sepharose column, showing five peaks (peaks 1–4 and sodin 5) with PNAG activity (arbitrary units). (**B**) SDS-PAGE analysis of 194–201 fractions (5.0 μg) from sodin 5 obtained after cation exchange chromatography (**A**). M, molecular weight markers. SDS-PAGE in the presence of β-mercaptoethanol was carried out in 12% polyacrylamide separating gel and then stained with Coomassie brilliant blue.

The crude extract from *S. soda* seeds, obtained by homogenizing seeds (50 g) in 500 mL of phosphate-buffered saline, pH 7.2, possessed the ability to depurinate the hsDNA substrate (data not shown) [38]. Thus, to ascertain the presence of type-1 RIPs, the total crude extract was subjected to acid precipitation following a protocol for the extraction of basic proteins [34]. The supernatant was subjected to step-wise cation exchange chromatography, and eluted basic proteins were separated by gel-filtration. Pooled fractions with a molecular weight around 29 kDa were further subjected to cation exchange chromatography on the CM-Sepharose column, eluted with a linear NaCl gradient (0–0.17 M) in 5 mM Na-phosphate buffer. As shown in Figure 1A, five protein peaks with PNAG activity were detected, confirming the presence of various PNAG-enzymes (isoforms), which is a common feature in plant RIP expression [15].

In this framework, we decided to first characterize the main protein peak, eluted at a higher ionic strength (peak 5). In particular, the fractions from 194 to 201, corresponding to the main peak (hereafter, sodin 5), were evaluated by SDS-PAGE, showing a single protein band with an electrophoretic migration of ~29 kDa (Figure 1B). Thus, the fractions were pooled, dialyzed and used for further enzymatic and structural characterization.

The purification yield obtained by this procedure was of about 2.9 ± 0.15 mg/100 g of seeds for sodin 5.

### 2.2. Enzymatic and Structural Features of Sodin 5

**Figure 2:**
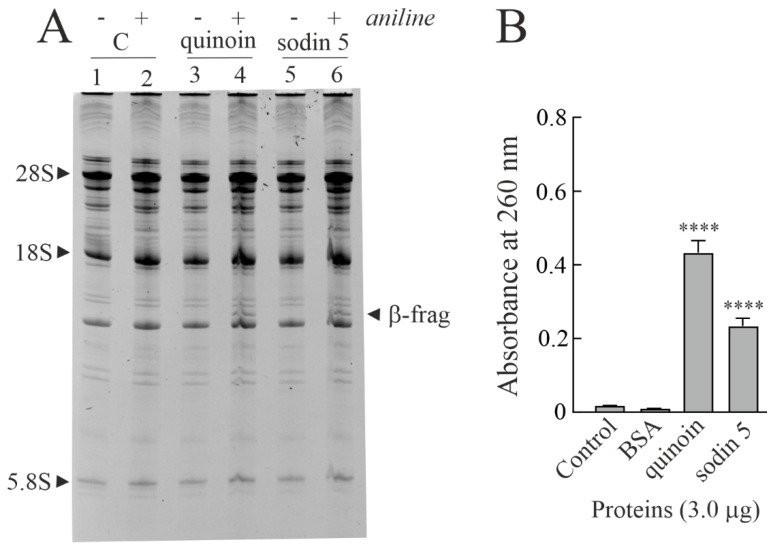
(**A**) rRNA N-glycosylase activity on rabbit ribosomes. Quinoin from *C. quinoa* seeds (3.0 µg; lanes 3 and 4) as a positive control and sodin 5 (3.0 µg; lanes 5 and 6) were incubated with ribosomes. Then, rRNA was extracted, treated with acid aniline and separated as described in the Materials and Methods section. (+) and (−) indicate with and without aniline treatment. ‘β-frag’ indicates the position of Endo’s fragment released by the aniline treatment of rRNA from rabbit ribosomes. (**B**) Polynucleotide:adenosine glycosylase activity of BSA (negative control) or quinoin and sodin 5 type-1 RIPs. Proteins (3.0 µg) were assayed on salmon sperm DNA as described in the Materials and Methods section. The mean results ± SD of three experiments performed in triplicate are reported. The data were compared to the control and analyzed by one-way ANOVA with Dunnett’s post hoc test (****, *p* < 0.0001).

In order to ascertain that the in vitro protein synthesis inhibition and the PNAG activity of sodin 5 were due to N-β-glycosylase action (characteristic enzymatic hallmark of RIPs from plants), we tested the β-fragment release by incubating the protein with rabbit ribosomes (Endo’s assay) [32]. As shown in Figure 2A, sodin 5 is able to deadenylate rRNA from rabbit reticulocyte lysate, releasing the β-fragment after aniline treatment. Furthermore, as demonstrated by the PNAG activity assay, sodin 5 is ~1.8-fold less active than quinoin, a type-1 RIP isolated from *Chenopodium quinoa* seeds and used as a reference PNAG-enzyme (Figure 2B) [39].

In addition, sodin 5 purified from *S. soda* seeds inhibited protein synthesis in a rabbit reticulocyte lysate system, with an IC_50_ value of 4.83 pM (0.14 ng/mL). This value is similar to that of quinoin (IC_50_= 5.08 pM; 0.15 ng/mL) and ~7.7-fold lower than that of saporin S6 (IC_50_ = 37 pM; 1.09 ng/mL) type-1 RIP isolated from *Saponaria officinalis* seeds [40]. On the other hand, the IC_50_ value of sodin 5 is of keen interest considering that type-1 RIPs have values of IC_50_ between 10 and 4000 pM [41].

**Figure 3:**
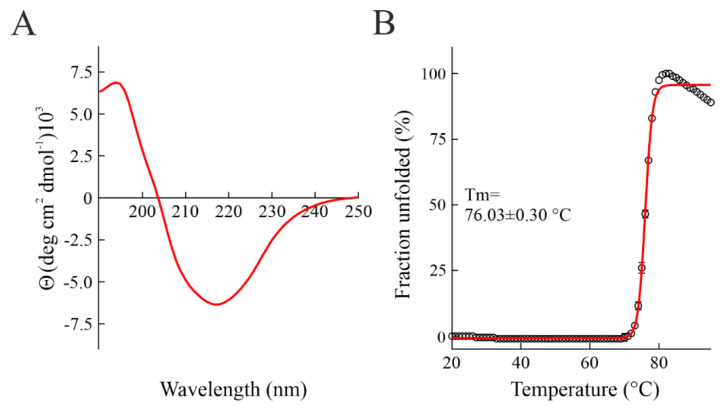
(**A**) Far-UV CD spectrum of sodin 5. (**B**) Thermal denaturation profile of sodin 5 (concentration: 0.15 mg mL^−^^1^). The fraction unfolded at 278 nm is plotted as a function of temperature. The red line represents fit curve.

The high amount of sodin 5 obtained from seeds of *S. soda* allowed us to perform a study on its secondary structure by Circular Dichroism (CD-) analysis. The far UV CD-spectrum of sodin 5 suggested that the periodic secondary structure of the protein is partially dominated by the α-helix, with a predicted percentage of ~30% (~25% β-strand) (Figure 3A). Therefore, these data show that sodin 5 has a secondary structure content similar to other RIPs, sharing a common 3D fold (RIP fold) consisting of a β-sheet N-terminal domain and an α-helix-rich C-terminal domain [42,43]. Subsequently, the thermal denaturation curve of sodin 5 was obtained using UV-spectroscopy by measuring the increment of absorbance at 278 nm, increasing the temperature. The melting temperature (Tm) of sodin 5 was 76.03 ± 0.30 °C (Figure 3B). The thermal unfolding curve at pH 7.2 shows that this type-1 RIP is a highly stable protein. In particular, the Tm value of sodin 5 is higher than that of both quinoin (68.2 °C [39]) and saporin S6 (58.0 °C) [44]. Both quinoin and saporin S6 have been isolated from Caryophyllales, the same plant order of *S. soda*.

Finally, considering that several type-1 RIPs are N-glycosylated, a specific analysis for glycoproteins detection was carried out. When analyzed by SDS-PAGE and sugar staining, sodin 5 appeared to be non-glycosylated (Figure S1).

### 2.3. Minor Forms of Type-1 RIPs from Salsola soda Seeds

To obtain information on minor peaks 1–4 (Figure 1A), showing PNAG activity and eluted at a lower ionic strength with respect to sodin 5, the fractions of each protein peak were analytically re-chromatographed by FPLC on an AKTA Purifier System from cation exchange chromatography using a Source 15S PE 4.6/100 column (Figure S2A). Each eluted peak displayed a single band of ~29 kDa by SDS-PAGE analysis (Figure S1A).

The pooled fractions of peaks 1–4, (hereafter sodins 1–4) were able to release the β-fragment similarly to sodin 5 as a consequence of the RIPs action (Figure S3). Therefore, sodins 1–4, with a molecular weight of ~29 kDa and specific N-β-glycosylase activity, can be considered type-1 RIPs. Moreover, among type-1 RIPs from *S. soda* seeds, sodin 1 displayed the higher PNAG activity (~2.3-fold more active than sodin 5), while sodins 2, 3 and 4 are, respectively, ~1.3-, 2.0- and 1.8-fold less active than sodin 5 (Figure S2B). These data agreed with the documented different ability of type-1 RIPs to deadenylate nucleic acid substrates [3].

In addition, the specific analysis for glycoproteins detection after SDS-PAGE was carried out. Similar to sodin 5, the analysis shows that these enzymes are non-glycosylated (Figure S1B).

The purification yield obtained by this procedure was of about 0.37 ± 0.01, 0.45 ± 0.02, 0.47 ± 0.01 and 0.67 ± 0.02 mg/100 g of seeds for sodins 1–4, respectively.

### 2.4. Type-1 RIPs from Edible Leaves and Roots of Salsola soda

**Figure 4:**
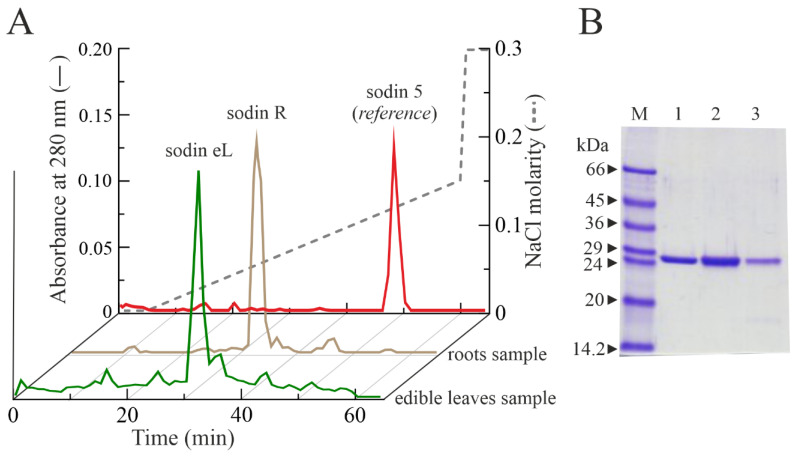
(**A**) Elution profile after FPLC on an AKTA Purifier System from cation exchange chromatography using a Source 15S PE 4.6/100 column, showing a single protein peak from *S. soda* roots and edible leaves, named sodin R and sodin eL, respectively. The elution profile of sodin 5 from *S. soda* seeds was reported as a reference chromatographic profile. (**B**) SDS-PAGE analysis of fractions (5.0 μg) from sodin 5, sodin R and sodin eL (lanes 1, 2 and 3, respectively), obtained after Source 15S chromatography. M, molecular weight markers. SDS-PAGE in the presence of β-mercaptoethanol was carried out in 12% polyacrylamide separating gel and then stained with Coomassie brilliant blue.

In order to evaluate the RIPs distribution in the tissues of *S. soda*, the same protocol used for the purification of sodins from *S. soda* seeds was also applied on the edible leaves and roots of this plant. The approach, coupled with the detection of enzymatic activity, allowed for the purification of two different type-1 RIPs from *S. soda* edible leaves and roots, respectively. However, considering the lower number of raw basic proteins, after cation step-wise chromatography and gel-filtration (see Section 4.2), the pools of basic proteins from the edible leaves and roots with a molecular weight of 29 kDa were separately subjected to analytical cation exchange chromatography using a Source 15S PE 4.6/100 column on AKTA Purifier System (Figure 4A).

The single protein peaks from edible leaves and roots were analyzed by SDS-PAGE to verify the purity and integrity. As reported in Figure 4B, both protein peaks showed the presence of a single band with an electrophoretic migration of ~29 kDa. These two-novel type-1 RIPs from edible leaves and roots were named sodin eL and sodin R, respectively.

The purification yield obtained by this procedure was of about 17.5 ± 0.61 µg/100 g of edible leaves and 27.8 ± 0.87 µg/100 g of roots for sodin eL and sodin R, respectively, confirming the low number of type-1 RIPs in edible leaves and roots compared to the quantity found in seeds.

In addition, the specific analysis for glycoproteins detection after SDS-PAGE shows that these enzymes are non-glycosylated, like both sodin 5 and sodins 1–4 from *S. soda* seeds (Figure S4).

**Figure 5:**
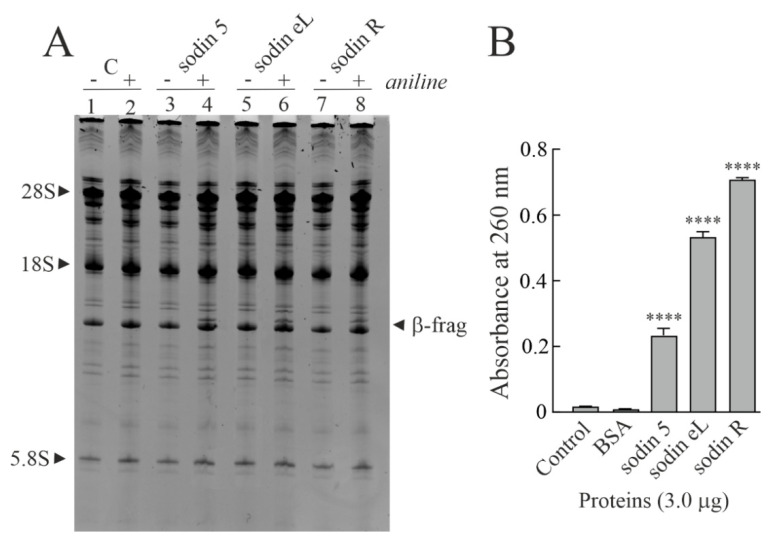
(**A**) rRNA N-glycosylase activity assayed on rabbit ribosomes. Sodin 5 (3.0 μg; lanes 3 and 4) as a positive control and sodin eL (3.0 μg; lanes 5 and 6) or sodin R (3.0 μg; lanes 7 and 8) were incubated with ribosomes. Then, rRNA was extracted, treated with acid aniline and separated as described in the Materials and Methods section. (+) and (−) indicate with and without aniline treatment. ‘β-frag’ indicates the position of Endo’s fragment released by the aniline treatment of rRNA from rabbit ribosomes. (**B**) Polynucleotide:adenosine glycosylase activity of BSA (negative control) or sodin 5, sodin eL and sodin R type-1 RIPs. Proteins (3.0 μg) were assayed on salmon sperm DNA as described in the Materials and Methods section. The mean results ± SD of three experiments performed in triplicate are reported. Data were compared to the control and analyzed by one-way ANOVA with Dunnett’s post hoc test (****, *p* < 0.0001).

Finally, the N-β-glycosylase action (characteristic enzymatic hallmark of RIPs from plants) of sodin eL and sodin R has been tested. As shown in Figure 5A, both proteins release the β-fragment by incubating the protein with rabbit ribosomes (Endo’s assay) following aniline treatment, similarly to sodin 5 from *S. soda* seeds. In addition, type-1 RIPs from *S. soda* roots and edible leaves also displayed PNAG activity. In particular, as shown in Figure 5B, sodin eL and sodin R displayed a PNAG activity that was ~2.2- and 2.9-fold higher than that of sodin 5.

**Figure 6:**
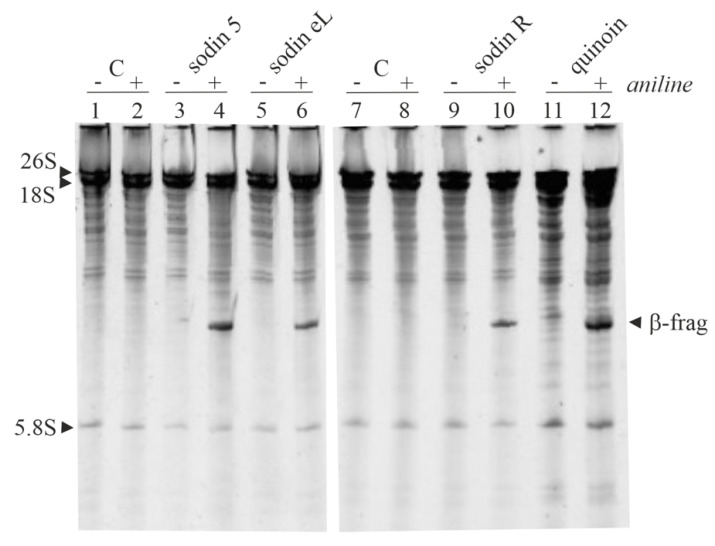
rRNA N-glycosylase activity assayed on yeast ribosomes. rRNA N-glycosylase activity was analyzed as reported in the Materials and Methods section. Each lane contained 5 µg of RNA isolated from either untreated (control) or RIP-treated ribosomes from yeast. (+) and (−) indicate with and without aniline treatment. ‘β-frag’ indicates the position of Endo’s fragment released by the aniline treatment of rRNA from yeast ribosomes.

In addition, since the rRNA N-glycosylase activity might play a role in plant defense, we assayed the effect of sodin 5, sodin eL and sodin R, as well as quinoin, on ribosomes from yeasts (*Saccharomyces cerevisiae*) homologous to ribosomes from the putative fungal pathogens of plants. As shown in Figure 6, these RIPs displayed rRNA N-glycosylase activity on yeast ribosomes, as indicated by the release of a diagnostic β-fragment identical to that reported for BE27 from *Beta vulgaris* L. (sugar beet) and type-1 RIPs from *Phytolacca dioica* L. [45,46].

Finally, sodin eL and sodin R inhibited protein synthesis in a rabbit reticulocyte lysate system, with IC_50_ values of 79.31 pM (2.3 ng/mL) and 65.52 pM (1.9 ng/mL), respectively. These values are ~15-fold higher with respect to the IC_50_ of sodin 5 isolated from *S. soda* seeds.

### 2.5. Cytotoxic Effects of Sodins from S. soda Tissues in Cell Cultures

RIPs are cytotoxic toward several human cell lines (normal and malignant), although type-1 RIPs are usually less cytotoxic than type-2 RIPs due to the lack of a B-chain, which improves the entry of the A-chain in the cells. Indeed, typical IC_50_ values of toxic type-2 RIPs on cultured animal cells are in the range of 0.3–17,000 pM, while IC_50_ values of type-1 RIPs are in the range of 170–3300 nM [41]. Thus, we decided to verify the cytotoxic effects of sodins or quinoin on both HeLa and COLO 320 cell lines.

Table 1 lists the IC_50_ values (concentration of protein causing the death of 50% of cells) of sodin 5, sodin eL and sodin R from *S. soda* seeds, edible leaves and roots, respectively, compared with the IC_50_ of quinoin. Type-1 RIPs from both *S. soda* tissues and *C. quinoa* seeds were toxic to HeLa and COLO 320 cells, exhibiting IC_50_ values ranging from 0.41 to 1200 nM.

**Figure 7:**
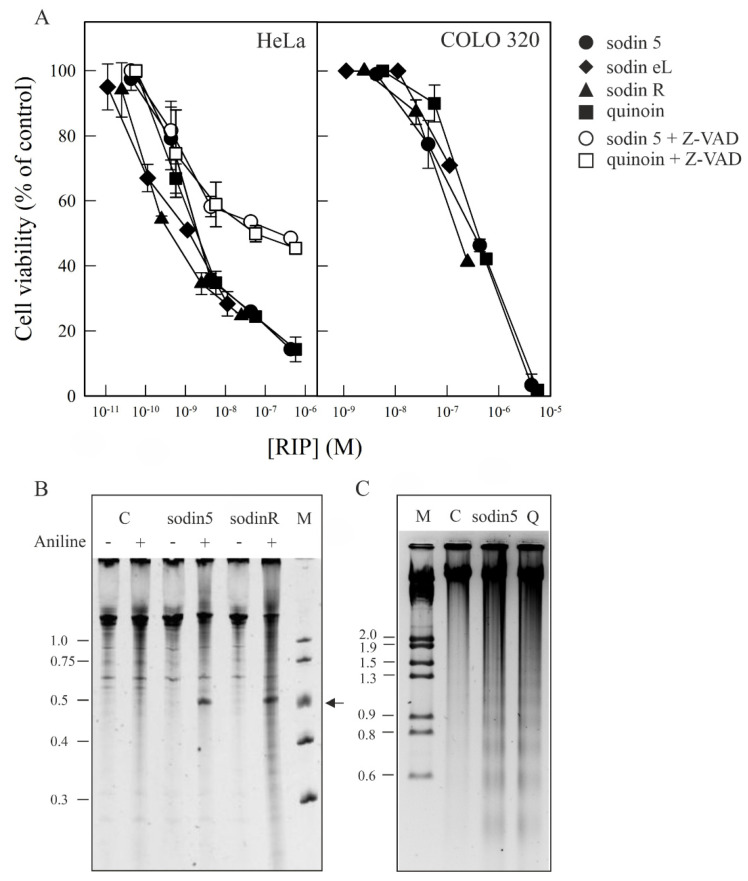
Induction of cytotoxicity and apoptosis on HeLa and COLO 320 cells by sodins and quinoin (**A**). Effect of sodins or quinoin on the viability of HeLa (left panel) and COLO 320 (right panel) cells. Cells were grown in RPMI 1640 medium and incubated with different type-1 RIP concentrations for 48 h (HeLa) and 72 h (COLO 320), and cell viability was evaluated by a colorimetric assay, as indicated in Section 4.6 of the Materials and Methods section. To investigate the effect of Z-VAD on the viability of HeLa cells, the cells were preincubated for 3 h with Z-VAD and then incubated with different concentrations of sodin 5 or quinoin for 48 h, and cell viability was evaluated. Data represent the mean ± SD of two experiments performed in duplicate. (**B**) rRNA N-glycosylase activity of sodin 5 and sodin R on RNA from HeLa cells. rRNA N-glycosylase activity was evaluated as reported in the Materials and Methods section. Each lane contained 2.0 μg of RNA isolated from either untreated cells (C, control) or cells incubated with 8 nM of sodin 5 or 5 nM of sodin R for 48 h. The arrow indicates the RNA fragment released as a result of RIP action upon the acid aniline treatment. Numbers indicate the size of the standards (M) in nucleotides. (**C**) Effect of sodin 5 and quinoin on internucleosomal DNA fragmentation. COLO 320 cells were incubated in the absence (C, control) or presence of 0.4 μM of sodin 5 or 0.6 μM of quinoin (Q) for 72 h. The DNA was isolated, and 4.0 μg was electrophoresed, as indicated in Section 4.7. The numbers indicate the corresponding size of the standards (M) (λDNA HindIII/EcoRI) in Kb. (+) and (−) indicate with and without aniline treatment.

The most sensitive were HeLa cells, with IC_50_ values from 0.41 to 2.0 nM after 48 h of treatment, while COLO 320 cells have values between 160 - >120 nM after 72 h of treatment (Figure 7A). These data agree with those previously reported for other type-1 RIPs, such as type-1 RIPs isolated from *P. dioica*, which exhibit IC_50_ values ranging from 1.0 to 1000 nM against the same cell lines [45]. On the other hand, the cytotoxicity of sodin 5 and quinoin is similar, with IC_50_ values 10^3^-fold higher for COLO 320 cells with respect to Hela cells.

There are important differences in toxicity among type-1 RIPs based on their capability to reach the ribosomes of target cells. Based on the above studies, sodins and quinoin display a considerable toxicity against HeLa cells for type-1 RIPs. To see if sodin 5 and sodin R can reach and inactivate the ribosomes after being endocytosed, we analyzed the rRNA from HeLa cells after 48 h of RIP treatment. Figure 7B displayed that the ribosomes were depurinated, as proved by the detection of a diagnostic β-fragment following RNA treatment with acid aniline. Thus, both sodin 5 and sodin R can reach the ribosomes of target cells, inhibiting protein synthesis.

Several studies highlight that RIP cytotoxicity in the cells is associated with their ability to induce apoptosis [12]. Apoptosis might be a consequence of the ribotoxic stress induced by the RIP after entry into the cytosol, or both processes could run in parallel. Apoptosis is characterized by cell shrinkage, nuclear condensation, changes in the cell membrane and mitochondria, DNA fragmentation into 200 base oligomers and protein degradation by caspases. In this framework, in order to ascertain if the observed cytotoxic effects of both sodin 5 and quinoin were mediated via apoptosis, we evaluated the sensitivity to the pan-caspase inhibitor Z-VAD or the cleavage of chromosomal DNA into oligonucleosomal fragments, considered a late-stage apoptosis hallmark. In particular, HeLa cells were pre-treated and maintained in 100 μM Z-VAD for 48 h, and the cell viability was determined for different sodin 5 and quinoin concentrations. As shown in Figure 7A, the presence of Z-VAD improved cell survival. In particular, in the presence of Z-VAD, viability increased from 14 to 49% in 4.3 × 10^−7^ M sodin 5-treated cells and from 14 to 46% in 5.7 × 10^−7^ M quinoin-treated cells. On the other hand, when COLO 320 cells were treated for 72 h with RIP concentrations close to their IC_50_, the breakdown of the nuclear DNA into oligonucleosomal fragments was clearly observed (Figure 7C).

Overall, our data suggested that the apoptotic pathway was implicated in the cell death mediated by sodin 5 and quinoin, as already proved for other type-1 RIPs [41].

### 2.6. Effect of Sodin 5 and Quinoin on the Growth of P. digitatum

**Figure 8:**
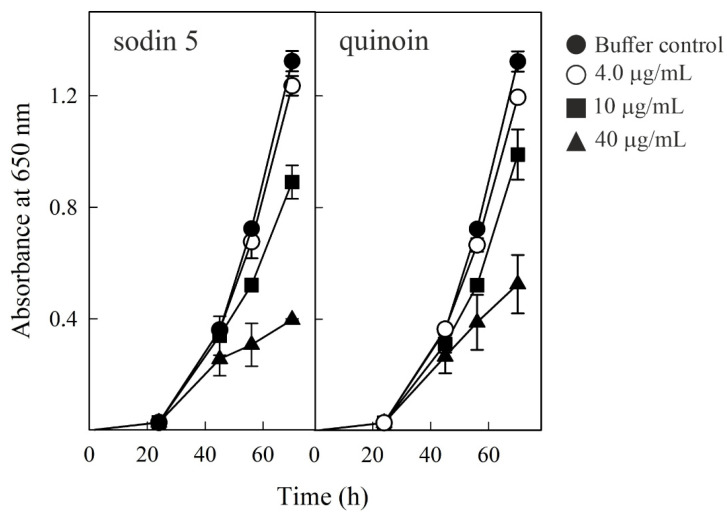
Antifungal activity of sodin 5 (left panel) and quinoin (right panel) against *Penicillium digitatum*, measured in a microtiter plate bioassay. Conidia of *P. digitatum* were grown in Potato Dextrose Broth (PDB) for 24 h before exposure to different RIP concentrations. Fungal growth was followed for 70 h and measured as an increase in absorbance at 650 nm. The curves represent the buffer control or different amounts (µg/mL) of both toxins. The mean results ± SE of two experiments performed in triplicate are reported.

A potential role for RIPs as plant defense proteins has been proposed based on their enzymatic activity, which can act by either inactivating pathogen ribosomes or their own ribosomes, causing cell death [27]. Antifungal activity has been attributed to several RIPs. In particular, a strong antifungal activity against *P. digitatum* has been described for the apoplastic type-1 RIP beetin 27 (BE27) from sugar beet and for the type-1 RIPs PD-S2 and dioicin 2 from *P. dioica* [45,47]. *P. digitatum* is a necrotrophic fungus responsible for the postharvest decay of citrus, an economically important crop worldwide. Therefore, we carried out experiments to evaluate the effects of sodin 5 from *S. soda* seeds and quinoin from *C. quinoa* seeds on the growth of *P. digitatum*. Thus, conidia of *P. digitatum* were grown in PDB medium for 24 h before exposure to different RIP concentrations, continuing the treatment for a further 46 h. As shown in Figure 8, sodin 5 and quinoin reduced the fungal growth in a concentration-dependent manner. Both RIPs induced a strong decrease in the growth at 40 µg/mL. Thus, 40, 10 and 4.0 µg/mL of sodin 5 resulted in 70%, 34% and 6% growth inhibition, respectively, after 70 h of growth. Similar results were obtained for quinoin, with 61%, 27% and 10% growth inhibition at the same concentrations. Sodin 5 and quinoin added from the beginning to the conidia as starting material inhibited fungal growth to the same extent as RIPs added at 24 h (once conidial germination occurred; data not shown), suggesting that sodin 5 and quinoin affect mycelial growth rather than conidial germination. As shown in Figure 2 and Figure 6, sodin 5 and quinoin exhibited rRNA N-glycosylase activities against mammalian and fungal ribosomes, respectively. Thus, the antifungal activities of sodin 5 and quinoin against *P. digitatum* could be mediated by the inhibition of protein synthesis together with the ability to cross the membrane and enter into the fungal cells, as has been postulated for other type-1 RIPs [45,47].

## 3. Conclusions

In conclusion, we have isolated seven type-1 RIPs from the different tissues of *S. soda* (‘agretti’ in Italian): five type-1 RIPs from seeds (sodins), one from edible leaves (sodin eL) and one from roots (sodin R). All these enzymes are able to release the β-fragment following incubation with rabbit or yeast ribosome and exhibit PNAG activity.

Sodin 5, the major form expressed in seeds (2.9 ± 0.15 mg/100 g of seeds), with respect to other type-1 RIPs from *S. soda* tissues, exhibits an α+β structure typical of type-1 RIPs with a high melting temperature (Tm = 76.03 ± 0.30 °C) and is non-glycosylated, as the other six sodins. Furthermore, sodin 5, sodin eL and sodin R show cytotoxic effects towards the HeLa and COLO 320 cell lines, inducing apoptosis. In addition, since fungi are among the most important plant pathogens, we tested the antifungal properties of both sodin 5 and quinoin against *P. digitatum*, finding that both RIPs possess concentration-dependent antifungal activity.

Overall, this research aims to revisit RIPs in edible plants in light of their possible use as antiviral, antifungal and antipathogenic tools in agri-food, overcoming the preconception about transgenic plants, as these enzymes are physiologically present in edible plants.

## 4. Materials and Methods

### 4.1. Materials

The chemicals for chromatography were previously reported [39,48,49]. Single-stranded salmon sperm DNA was obtained from Sigma-Aldrich (St. Louis, MO, USA). Quinoin from the seeds of *C. quinoa* and PD-L4 from the leaves of *P. dioica* were isolated as previously reported [34,39]. The nuclease-treated rabbit reticulocyte lysate system was purchased from Promega (Madison, WI, USA).

The medium and the other chemicals were from Sigma Chemical Co. (St. Louis, MO, USA). The RPMI 1640 medium, fetal bovine serum (FBS), penicillin, streptomycin and trypsin were purchased from GIBCO BRL (Barcelona, Spain). The Z-VAD-fmk (pan-caspase inhibitor carbobenzoxy-valyl-alanyl-aspartyl-[O-methyl]-fluoromethylketone) named Z-VAD was purchased from R&D Systems (Abingdon, UK).

Buffer A: 5 mM Na-phosphate, pH 7.2, containing 0.14 M NaCl; buffer B: 10 mM Na-acetate, pH 4.0; and buffer C: 5 mM Na-phosphate, pH 7.2.

### 4.2. Purification of Type-1 RIPs from Seeds, Roots and Edible Leaves of S. soda

Type-1 RIPs from *S. soda* were purified with the same protocol used for quinoin, type-1 RIP from *C. quinoa* seeds, as reported by Landi et al., 2021 [39]. Briefly, the crude extract in buffer A was first subjected to acid precipitation at pH 4.0 and cation step-wise chromatography using a SP-Streamline resin [column L × I.D. 20 cm × 30 mm, flow rate 3.0 mL/min; Cytiva, Buccinasco (MI) Italy]. Subsequently, the basic proteins, eluted with 1.0 M NaCl in buffer C, were gel-filtrated [HiLoad^®^ 26/60 Superdex^®^ column L × I.D. 60 cm × 26 mm, flow rate 2.5 mL/min (range 100–10 kDa); Cytiva] to separate the proteins by molecular weight, and then, basic proteins with a molecular weight of about 29 kDa were subjected to cation exchange chromatography on CM-Sepharose fast flow (Cytiva; column L × I.D. 25 cm × 16 mm) equilibrated in buffer C and eluted with a NaCl gradient up to 0.17 M (buffer C, 500 mL, buffer C containing 0.17 M NaCl, 500 mL; total volume 1 L using a peristaltic pump).

However, when the number of basic proteins after gel-filtration was lower (less than 200 µg), CM-Sepharose chromatography was replaced by FPLC on an AKTA Purifier System (Amersham Pharmacia; Milan, Italy) using a Source 15S PE 4.6/100 column, equilibrated in buffer C and eluted by a linear gradient from 0 to 50% of buffer C containing NaCl 0.3 M over 60 min (flow rate 1.0 mL/min). The same chromatographic step (Source 15S column) was carried out for minor forms of type-1 RIPs from the seeds of *S. soda* after cation exchange chromatography on the CM-Sepharose column.

### 4.3. Enzymatic Assays

#### 4.3.1. rRNA N-Glycosylase Activity of RIPs on Rabbit Ribosomes

The rRNA N-glycosylase assay was conducted as previously described [45]. Rabbit reticulocytes lysate (40 μL) was incubated with RIP (3.0 μg) at 37 °C for 1 h. After treatment, the RNA was extracted by phenolization, treated with 1 M aniline acetate (pH 4.5) and precipitated with cold ethanol. Purified RNA was analyzed by polyacrylamide gel in denaturing conditions [7 M urea/5% acrylamide (*w*/*v*)] and stained with ethidium bromide.

#### 4.3.2. rRNA N-Glycosylase Activity of RIPs on Yeast Ribosomes

The preparation of the 30,000 g (S30) supernatants from yeast was performed as described elsewhere [46]. The rRNA N-glycosylase activity was assayed in 50 μL samples of S30 supernatant from yeast, which was incubated with 5.0 µg of sodin 5, 0.7 µg of sodin eL, 1.5 µg of sodin R or 5.0 µg of quinoin for 1 h at 30 °C. After treatment, the RNA was extracted with phenol and treated with aniline for 10 min at 23 °C. The RNA samples were separated on a polyacrylamide gel in denaturing conditions [7 M urea/5% acrylamide (w/v)] and stained with Gel Red nucleic acid staining [50].

#### 4.3.3. Polynucleotide: Adenosine Glycosylase Activity on Salmon Sperm DNA

The adenine release was measured as previously reported [45], incubating salmon sperm DNA (10 μg) with RIPs (3.0 μg) in 300 μL 50 mM magnesium acetate (pH 4.0) containing 100 mM KCl, at 30 °C for 1 h. After incubation, the DNA was precipitated with cold ethanol and centrifuged. Adenine release was determined spectrophotometrically, reading the supernatant at 260 nm. On the other hand, to evaluate arbitrary units of PNAG activity on single fractions from *S. soda* seeds after CM-Sepharose chromatography, an equal volume was tested.

#### 4.3.4. Cell-Free Protein Synthesis Inhibition

The effect of RIPs on protein synthesis was determined through a coupled transcription-translation in vitro assay using a rabbit reticulocytes lysate system, as described elsewhere [51]. Samples of RIPs were diluted and added to the reaction mixture as previously described [51]. Three experiments were conducted in duplicate, and IC_50_ (concentration that inhibits 50% protein synthesis) values were calculated by linear regression.

### 4.4. Analytical Procedures

The proteins’ homogeneity was evaluated by SDS-PAGE with a Mini-Protean II (Bio-Rad; Milan, Italy) using a 6% stacking and 12% separating polyacrylamide gel under reducing conditions; a precision plus protein kit (Bio-Rad) was used as the reference proteins. The protein concentration was determined by a Pierce BCA Protein Assay Kit (Life Technologies Italia Fil., Monza, Italy). The glycosylation analysis was performed in gel after SDS-PAGE by using the Pro-Q™ Emerald 300 Glycoprot Probes Kombo (Life Technologies Italia). Glycosylated proteins were visualized by a ChemiDocTM XRS system.

### 4.5. Circular Dichroism and Thermal Stability Determination

The far-UV CD spectrum of sodin 5 was determined at 25 °C on a Jasco J-815 dichrograph [Jasco Europe, Cremella (LC) Italy]. A protein concentration of 0.15 mg/mL (5.15 μM) in 10 mM Na-phosphate, pH 7.2 (path-length quartz cuvette of 0.1 cm), was used for the far-UV spectrum measurements. DichroWeb (online analysis for protein Circular Dichroism spectra; http://dichroweb.cryst.bbk.ac.uk/html/home.shtml (accessed on 8 June 2022); [52]) was used to estimate the percentages of secondary structural elements.

Protein (~0.15 mg/mL) in 10 mM sodium phosphate, pH 7.2, was subjected to heat-induced denaturation, as previously reported [39].

### 4.6. Cell Viability Assays

The COLO 320 (human colon adenocarcinoma) and HeLa cell lines used in this study were obtained from the European Collection of Cell Cultures (ECACC). The cells were grown in RPMI 1640 medium (GIBCO BRL, Barcelona, Spain) supplemented with 10% fetal bovine serum (FBS), 100 U mL^−1^ penicillin and 0.1 mg mL^−1^ streptomycin under 5% CO_2_ at 37 °C. Cell viability was determined as previously reported [45]. The concentration of RIPs causing a 50% reduction in viability (IC_50_) was calculated by linear regression analysis. Sodin 5 and quinoin toxicity was also evaluated using HeLa cells pre-treated with 100 μM of the pan-caspase inhibitor Z-VAD. The reagent was added to cells 3 h before RIP administration, and the cell viability was determined for different RIP concentrations.

### 4.7. DNA Fragmentation Analysis

COLO 320 cells (1 × 10^6^/plate) were incubated for 72 h in the presence of RIP (~0.5 µM). After treatment, cells were harvested by centrifugation (1000× *g* for 5 min). The pellets were lysed in 50 mM Tris Cl, pH 8.0, containing 10 mM EDTA and 0.5% SDS, and the DNA was isolated following the manufacturer’s instructions [Genomic Prep Cells and Tissue DNA Isolation Kit (GE Healthcare, Madrid, Spain)]. DNA electrophoresis was carried out as previously reported [45].

### 4.8. Antifungal Activity Measurements

The growth inhibition assays of sodin 5 from *S. soda* seeds and quinoin from *C. quinoa* against *P. digitatum* were performed in 96-well microtiter plates. The conidia of *P. digitatum* (100 spores/well), obtained as indicated [47], were incubated at 26 °C in 150 μL PDB medium for 24 h to allow for conidia germination. The incubation was continued in the presence or in the absence of different RIP concentrations for a further 46 h. Fungal growth was followed for 70 h and measured as an increase in absorbance at 650 nm. Fungal growth was monitored spectrophotometrically using a microtiter plate reader (ELISA reader Multiskan) after 0, 24, 45, 56 and 70 h of incubation. The absorbance of cultures without cells was subtracted as the background.

## Figures and Tables

**Table 1:** Cytotoxicity of sodins and quinoin. HeLa or COLO 320 cells were grown in RPMI 1640 medium and incubated with different RIP concentrations for 48 or 72 h, and cell viability was evaluated by a colorimetric assay, as indicated in Section 4.6 of the Materials and Methods section. When reported, cells were pre-treated with Z-VAD for 3 h (see Materials and Methods) and then incubated with different RIP concentrations. Data represent the mean of three experiments performed in triplicate.

Type-1 RIP	HeLa 48 h		COLO 48 h	COLO 72 h
	--	+Z-VAD Pretreatment	--	
quinoin	1.9 × 10^−9^	1.0 × 10^−7^	1.0 × 10^−6^	3.9 × 10^−7^
sodin 5	2.0 × 10^−9^	2.5 × 10^−7^	1.2 × 10^−6^	3.3 × 10^−7^
sodin eL	1.3 × 10^−9^	--	--	>1.2 × 10^−7^
sodin R	4.1 × 10^−10^	--	--	1.6 × 10^−7^

## Data Availability

The data presented in this study are available in this article.

## Supplementary Materials

The following supporting information can be downloaded at: https://www.mdpi.com/article/10.3390/toxins14080566/s1, Figure S1: SDS-PAGE analysis and in-gel staining for sugars of sodins isolated from *S. soda* tissues; Figure S2: Protein purification and polynucleotide:adenosine glycosylase activity of sodins 1–4; Figure S3: rRNA N-glycosylase activity of sodins from *S. soda* seeds assayed on rabbit ribosomes; Figure S4: staining for sugars of sodins isolated from *S. soda* tissues after SDS-PAGE.

## Abbreviations

CD, circular dichroism; IC_50_, concentration that inhibits 50% of protein synthesis or reduces 50% of cell viability; PDB, potato dextrose broth; PNAG, polynucleotide:adenosine glycosylase; RIPs, ribosome-inactivating proteins; SRL, sarcin ricin loop; Tm, melting temperature.
