# Supplementary material for: Isolation, Characterization and Biological Action of Type-1 Ribosome-Inactivating Proteins from Tissues of Salsola soda L
Source: Toxins (Basel). 2022 Aug 19;14(8):566. doi: 10.3390/toxins14080566 (PMC9412391; doi:10.3390/toxins14080566)
Supplement: Supplementary file 1 [file toxins-14-00566-s001.zip › toxins-1853975-supplementary.pdf]

Supplementary Materials

Isolation, Characterization and Biological Action of Type-1 Ribosome-Inactivating Proteins from Tissues of *Salsola soda* L.

Nicola Landi, Sara Ragucci, Lucía Citores, Angela Clemente, Hafiza Z. F. Hussain, Rosario Iglesias, José M. Ferreras and Antimo Di Maro

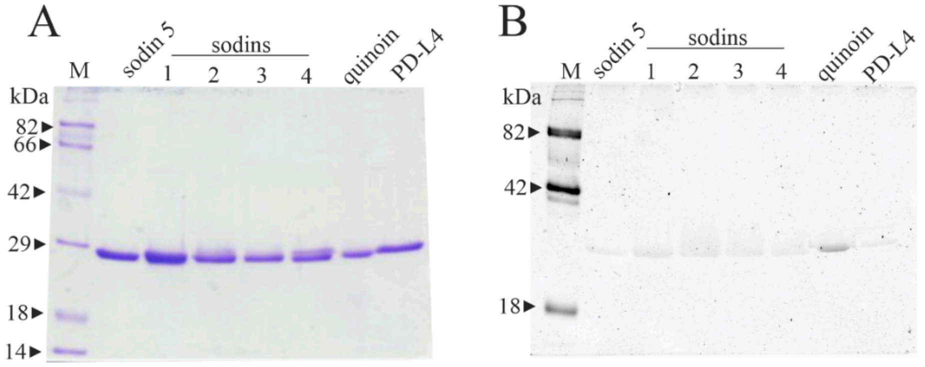

Figure S1. (**A**) SDS-PAGE analysis (**B**) and in gel staining for sugars of sodins, isolated from *S. soda* tissues. 3.0 µg (0.1 nmole) of sodin 5 (major form from *S. soda* seeds), sodins 1-4 (lanes 1, 2, 3 and 4), quinoin (N-glycosylated type-1 RIP from *C. quinoa* seeds) and PD-L4 (non-glycosylated type-1 RIP from *P. dioica* leaves) were loaded, respectively. M, Coomassie blue (left panel) and CandyCane™ glycoproteins (right panel) molecular weight standards, respectively. SDS-PAGE was carried out on a 12% polyacrylamide separating gel in the presence of β-mercaptoethanol, followed by in-gel glycan detection using the Pro-Q Emerald 300 glycoprotein staining kit. Stained glycoproteins were visualised by UV trans-illumination.

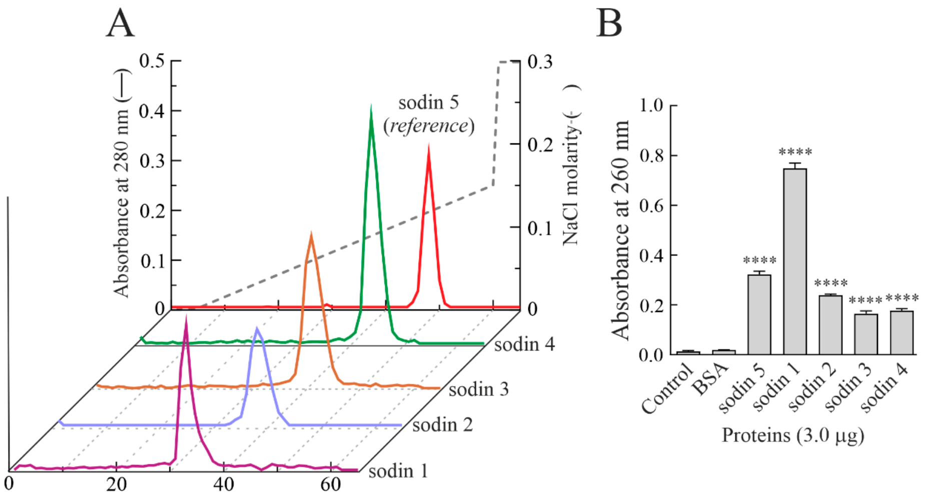

Figure S2. Protein purification and polynucleotide:adenosine glycosylase activity of sodins 1-4. (**A**) Elution profile after re-chromatography FPLC on an AKTA Purifier System from cation exchange chromatography using a Source 15S PE 4.6/100 column, showing peaks 1-4 (sodins 1-4) with the ability to release the β-fragment. The elution profile of sodin 5 was reported as reference. (**B**) Polynucleotide:adenosine glycosylase activity of BSA (negative control) or sodins 1-5 type-1 RIPs. Proteins (3.0 µg) were assayed on salmon sperm DNA as described in Materials and methods. The mean results ± SD of three experiments performed in triplicate are reported. Data were compared to control and analysed by One-way ANOVA with Dunnett's post hoc test (****, $p < 0.0001$).

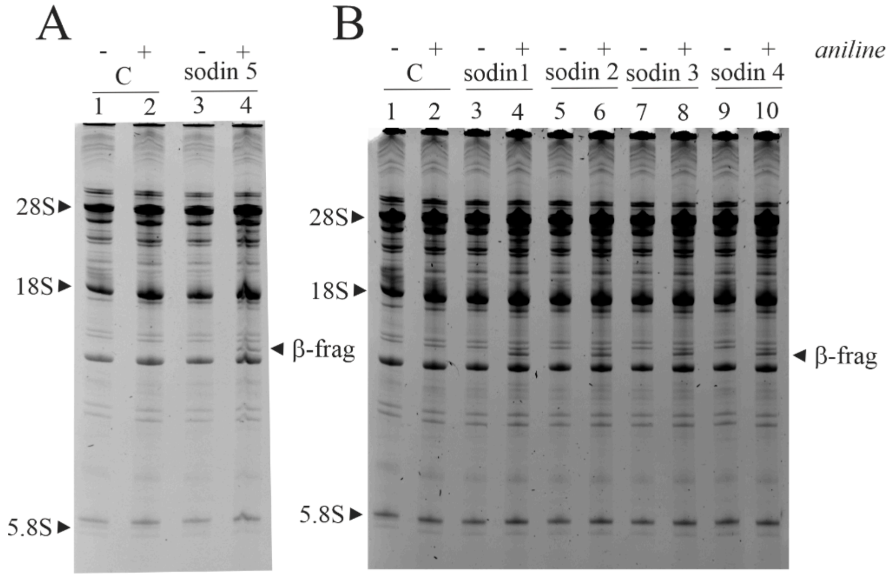

Figure S3. rRNA N-glycosylase activity of sodins from *S. soda* seeds assayed on rabbit ribosomes. Ribosomes were incubated with (**A**) 3.0 µg of type-1 RIP sodin 5, (lanes 3 and 4) as positive control and (**B**) 3.0 µg of sodins 1-4 (lanes 3-10, with or without aniline treatment). Following incubation, rRNA was extracted, treated with acid aniline and separated as reported in Materials and methods section. (+) and (-) indicate with and without aniline treatment. 'β-frag' indicates the position of Endo's fragment released by aniline treatment of rRNA from rabbit ribosomes.

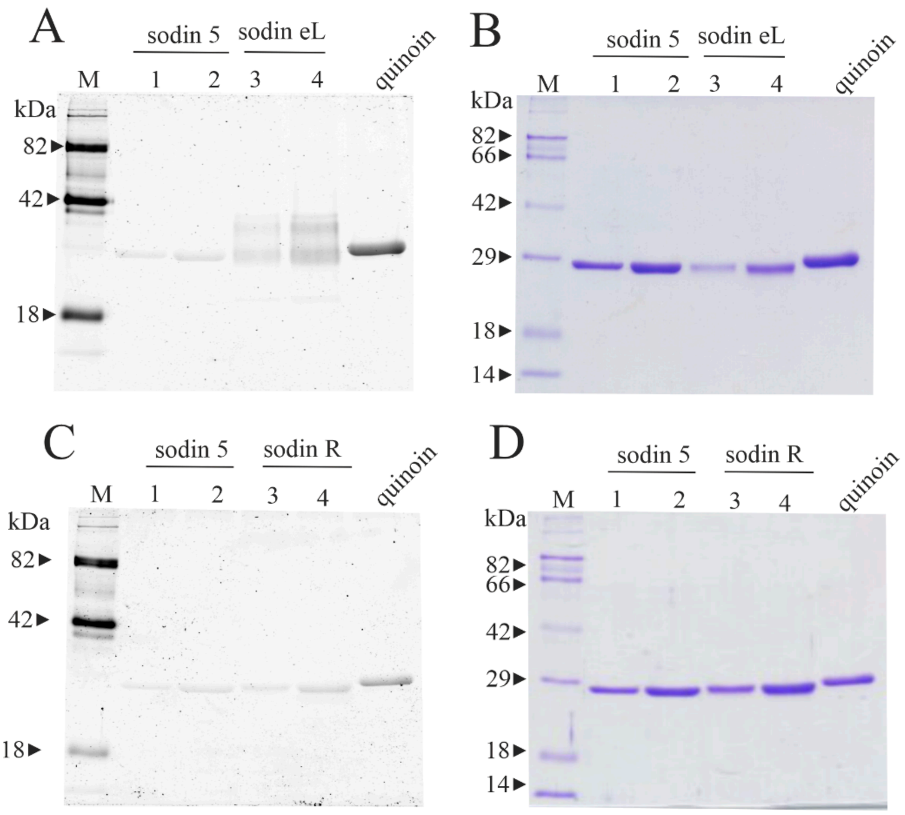

Figure S4. Staining for sugars of sodins isolated from *S. soda* tissues after SDS-PAGE. (**A**) and (**B**) 1.5 and 3.0 µg (0.05 and 0.1 nmole, respectively) of sodin 5 (lanes 1 and 2, respectively) and sodin eL (type-1 RIP from *S. soda* edible leaves; lanes 3 and 4, respectively) as well as 3.0 µg (0.1 nmole) of quinoin were loaded, respectively. (**C**) and (**D**) 1.5 and 3.0 µg (0.05 and 0.1 nmole, respectively) of sodin 5 (lanes 1 and 2, respectively) and sodin R (type-1 RIP from *S. soda* roots; lanes 3 and 4, respectively) as well as 3.0 µg (0.1 nmole) of quinoin were loaded, respectively. M, CandyCane™ glycoproteins (left panels) and Coomassie blue (right panels) molecular weight standards, respectively. SDS-PAGE was carried out on a 12% polyacrylamide separating gel in the presence of β-mercaptoethanol, followed by in-gel glycan detection using the Pro-Q Emerald 300 glycoprotein staining kit. Stained glycoproteins were visualised by UV trans-illumination.
